# Editorial: Improving the sustainability of winegrape vineyards during climate change

**DOI:** 10.3389/fpls.2023.1314923

**Published:** 2023-10-26

**Authors:** Balazs Barna, Nazareth Torres, Suzy Y. Rogiers

**Affiliations:** ^1^ Plant Protection Institute, Research Centre for Agriculture, Hungarian Research Network (HUN-REN), Budapest, Hungary; ^2^ Department of Agronomy, Biotechnology and Food Science, Public University of Navarre, Pamplona, Spain; ^3^ Institute for Multidisciplinary Research in Applied Biology (IMAB-UPNA), Public University of Navarre, Pamplona, Spain; ^4^ NSW Department of Primary Industries, Wollongbar Primary Industries Institute, Wollongbar, NSW, Australia

**Keywords:** high temperature, solar radiation, terroir, grape quality, flavonoids

Temperatures are increasingly becoming warmer due to an increase in greenhouse gas (GHG) emissions by anthropogenic activities and extreme events are becoming the ´new norm´. Even under very low GHG emissions in future scenarios, temperatures are estimated to remain elevated until at least 2,100. This tendency challenges the sustainability of crops. Grapevine is quite tolerant to environmental stresses, however, climate change related factors affect directly to grapevine cultivation, given their effect on `terroir´. Terroir is a highly important concept in viticulture because it relates the sensory attributes of wine to the environmental conditions in which the grapes are grown (Van Leeuwen and Seguin, 2006). In order to maintain proper growing temperatures, vineyards are currently experiencing an expansion towards higher altitudes. At these high altitudes, the environments are characterized by high thermal amplitudes and great solar radiations, especially ultraviolet-B (UV-B). The review by Arias et al. summarizes the environmental contribution of global high altitude-related climatic variables to the grapevine physiology and wine composition, which helps a better evaluation of the possible establishment of vineyards at high altitude in climate change scenarios.

Globally, vineyards cover approximately 7.4 M ha. The potential for carbon (C) storage in vineyards is of great interest to offset greenhouse gas emissions and mitigate the effects of climate change. To measure the potential for C storage in vineyards under varying sustainable soil management practices, Zumkeller et al. calculated the net ecosystem carbon balance (NECB) of three cover crops [perennial grass (*Poa bulbosa* hybrid cv. Oakville Blue); annual grass (barley, *Hordeum vulgare*); resident vegetation (natural weed population)] under conventional tillage (CT) and no-till (NT) management. Their results revealed that site characteristics, namely, soil texture and climate, were key determinants of the C storage potential of vineyards in Mediterranean climates such as those found in coastal and inland California wine grape production regions.

Traditional management practices in vineyards encouraged solar radiation exposure in wine grape fruit zone to promote flavonoid and aroma development. However, increasingly frequent heat wave events in hot grape growing regions are threating wine quality due to degradation of these desirable compounds in the grape berry. Thus, to maintain desirable chemical and aromatic properties in red wines, heat mitigation strategies need to be implemented in the vineyard. Marigliano et al. determined the cascading effects of partial solar radiation exclusion in the vineyard on resultant wine flavonoid and aroma composition. Overhead shade film D4 produced wines with improved color intensity, TPI, anthocyanin and flavonol profiles compared to C0 wines. Similarly, D4 wines were fruitier and with pleasant aroma profile compared to C0 wines.

While warm and cool growing regions may have different practical concerns related to climate change, they both experience altered berry and must composition and potentially reduced desirable wine characteristics and market value. Storms, drought and uncertain water supplies combined with excessive heat not only depress vine productivity through altered physiology but can have direct consequences on the fruit. Sunburn, shrivelling and altered sugar-flavour-aroma balance are becoming more prevalent while bushfires can result in smoke taint. Furthermore, distorted pest and disease cycles and changes in pathogen geographical distribution have altered biotic stress dynamics that require novel management strategies. A multipronged approach to address these challenges may include alternative cultivars and rootstocks or changing geographic location. In addition, modifying and incorporating novel irrigation regimes, vine architecture and canopy manipulation, vineyard floor management, soil amendments and foliar products such as antitranspirants and other film-forming barriers are potential levers that can be used to manage the effects of climate change (Rogiers et al.).

Under the scenario of climatic change, radiative excess, correlated to the increase in temperature, can subject the photosynthetic apparatus to a condition of light saturation and cause a drastic reduction in photochemical efficiency, giving rise to chronic photoinhibition phenomena. The objective of the study of Miciche et al. was to determine how artificial canopy shading affects the vine’s vegetative growth and the ripening processes of *Vitis vinifera* cv. Nero d’Avola during the 2019-2020 vegetative seasons. Three treatments were established: a shade treatment with a green net (shade factor 27%), a shade treatment with a white net (shade factor 32%), and untreated vines naturally exposed to light radiation. Artificial shading, applied at full fruit set, interfered with the microclimate of the vines, causing partial effects on the grape ripening processes. At harvest, no significant differences were found between the treatments in terms of sugars, while shading treatments increased must acidity and decreased pH. Results obtained on the vegetative parameters, suggest that the shading treatment delays leaf fall, with potentially positive effects on starch accumulation in the perennial reserve organs, potentially to be exploited during the following season’s spring root and shoot growth. Shading significantly reduced berry size, with obvious consequences on bunch weight and yield per vine. In 2020, all the phenological phases of the shaded plants were delayed. The total anthocyanin content was not changed by the shading treatment. The results obtained confirm the importance of net coverage on the microclimate of the vines, vegetative-productive activity, and grape quality. From this point of view, the net covering technique can be a tool for controlling grape ripening dynamics in the context of climate change. The Australian wine industry is already implementing site-specific adaptive measures to deal with unpredictable weather, droughts and rising temperatures. Growers are conscious of their environmental footprint, their long-term sustainability, and are balancing these visions with economic viability. The increasing awareness of sustainable management practises will result in overall improvements in maintaining an environmental equilibrium and promote soil health for future generations. As they become more economical and user-friendly, technical innovations will be readily adopted and integrated with more traditional approaches to vineyard management. However, long-term field trials are required to fine-tune these adaptive strategies to particular situations. Additionally, the inclusion of stakeholders in a co-design framework for future R&D will accelerate the adoption of these mitigation strategies.

## Author contributions

BB: Writing – review & editing. NT: Writing – review & editing. SR: Writing – review & editing.
